# A Survey of Acute Pain Service Structure and Function in United States Hospitals

**DOI:** 10.1155/2011/934932

**Published:** 2011-04-03

**Authors:** Dawood Nasir, Jo E. Howard, Girish P. Joshi, Gary E. Hill

**Affiliations:** ^1^Department of Anesthesiology and Pain Management, The University of Texas Southwestern Medical Center, 5323 Harry Hines Boulevard, Dallas, TX 75390-9068, USA; ^2^Parkland Health and Hospital System, Dallas, TX 75235, USA

## Abstract

Although the number of U.S. hospitals offering an acute pain service (APS) is increasing, the typical structure remains unknown. This survey was undertaken to describe the structure and function of the APS in U.S. hospitals only. We contacted 200 non-teaching and 101 teaching U.S. hospitals. The person in charge of postoperative pain management completed and returned the survey. Seventy-four percent of responding hospitals had an organized APS. An APS was significantly more formally organized in academic/teaching hospitals when compared to non-teaching hospitals. Pain assessments included “pain at rest” (97%), “pain on activity” (63%), and reassessment after pain therapy intervention (88.8%). Responding hospitals utilized postoperative pain protocols significantly more commonly in teaching hospitals when compared to non-teaching and VA hospitals. Intravenous patient controlled analgesia (IV-PCA) was managed most commonly by surgeons (75%), while epidural analgesia and peripheral nerve block infusions were exclusively managed by anesthesiologists. For improved analgesia, 62% allowed RNs to adjust the IV-PCA settings within set parameters, 43% allowed RN adjustment of epidural infusion rates, and 21% allowed RN adjustment of peripheral nerve catheter local anesthetic infusion rates.

## 1. Introduction

Despite of improved understanding of pain mechanisms and development of new analgesics techniques [1], under-treatment of postoperative pain continues [2]. It is suggested that availability of acute pain service (APS) would allow use of evidence-based approach to pain management and reduce the variations in pain management as well as provide wider choice of analgesic techniques and increase accountability [3, 4]. Overall, APS would improve postoperative pain management and patient satisfaction. Although the presence of an APS represents a step forward in postoperative pain control, its structure and functions across the United States remains unclear. In addition, the source of funding for APS, which is critical in the current economic environment of cost containment in healthcare, remains unknown. Furthermore, the involvement of nurses with pain management including pain assessment and implementation of analgesic protocols (i.e., clinical pathways) remains unknown [5–8]. 

This survey was designed to examine the structure and function of the APS in three different types of hospitals (e.g., academic, community based, and veterans administration (VA)) ranging in size from less than 200 beds to over 1000 beds across the US. In addition, we set out to determine the sources of funding for APS as well as evaluate delegated nursing responsibilities and management of commonly used postoperative analgesic techniques (i.e., intravenous patient-controlled analgesia (IV-PCA), epidural analgesia, and peripheral nerve catheter infusions).

## 2. Methods

After approval by the institutional review board as an exempt research, a research assistant emailed a request to participate to 200 nonacademic hospitals selected from http://www.officialusa.com/stateguides/health/hospitals/index.html—a website listing of hospitals in the US. To be considered for this survey, the hospital web page must have included a “contact us” hyperlink. The research assistant then contacted the hospital and attempted to reach the physician in charge of the acute pain service. If there was no official APS physician, the research assistant requested the name and contact information of a health care provider in charge of postoperative pain control at the hospital. The cover letter and online survey (Figure 10) were emailed to the designated person for completion and return. A single followup call was made if the survey had not been submitted within one month. 

The research assistant also used a secondary means of recruitment by faxing the cover letter and survey to a listing of 101 teaching hospitals in the US with anesthesiology residency programs. The materials were directed to the anesthesiology department chairperson who either completed the survey or delegated it to another provider in charge of postoperative pain control. Data collection occurred from January through March 2009. 

The Fisher's exact probability test resulting in a two-tailed (two-sided) *P* value or an unpaired Student's *t*-test (when appropriate) was used to compare groups (hospital types) on the reported variables. *P* values of  .05 or less defined significance.

## 3. Results

### 3.1. Hospital Demographics

A total of 108 questionnaires were returned out of 301 requests for participation, yielding an overall response rate of 35.9%. Of these responses, 55 hospitals were university-based medical centers, 40 were nonteaching (private) hospitals and 13 were VA hospitals. The geographic distribution across the US yielded 30 responses from the South, 18 responses from the West, 26 from the Midwest, 22 responses from the Northeast, and 12 unspecified. The size of the hospitals varied: 21 hospitals with fewer than 200 beds (19.4%), 49 with 200–500 beds (45.4%), 34 with 501–1000 beds (31.5%), and 4 with more than 1000 beds (3.7%). 

**Figure 1:**
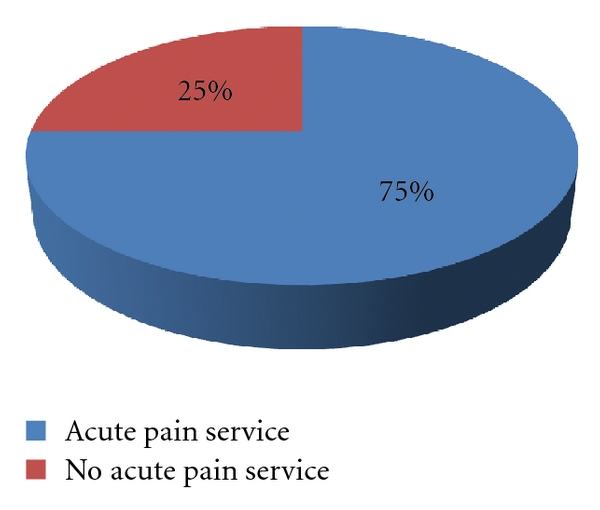
Percentage of responding hospitals with an organized acute pain service (APS).

Eighty one hospitals had an organized APS (75%), and 27 did not (25%) (Figure 1). The likelihood of an APS was directly correlated to hospital size: hospitals with >1,000 beds (100%), 501–1000 beds (93.7%), 200–500 beds (79%), and <200 beds (52.2%).

Responding university/academic hospitals reported a higher rate of an organized APS (96%) when compared to private hospitals (47%, *P* < .01), and VA Hospitals (69%, *P* < .05).

### 3.2. APS Demographics and Funding

**Figure 2:**
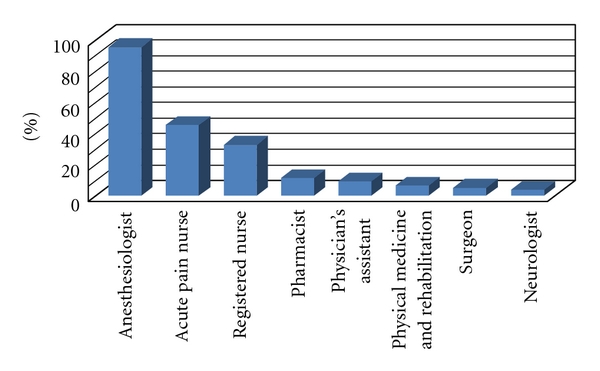
Percentage of APS personnel represented by different job descriptions, Anes (anesthesiologist), APN (acute pain nurse, nursing department member assigned primarily to APS, may be RN, PA, or nurse practitioner), Pharm (pharmacist), PA (physician assistant).

The personnel comprising the typical APS included anesthesiologists (95%), advanced practice nurses (APN, 45%), registered nurses (RN, 32.5%), pharmacists (11.3%), physician assistants (8.8%), physical medicine and rehabilitation (PMR) physicians (6.3%), surgeons (5%), neurologists (3.8%), and others (oncologists, social workers, and psychologists) (Figure 2). Seventy percent reported that that APS existed separately from the chronic pain service. The 75% (60 of 81) of the organized APS was funded by the anesthesia department (significantly greater for academic (88%), when compared to private hospitals (12%) and VA (0%), *P* < .01). In 25% of the reporting hospitals, funding for the APS came from the general budget, while no institution reported that the APS was funded by the surgery department. 

### 3.3. Pain Assessment and Reassessment

**Figure 3:**
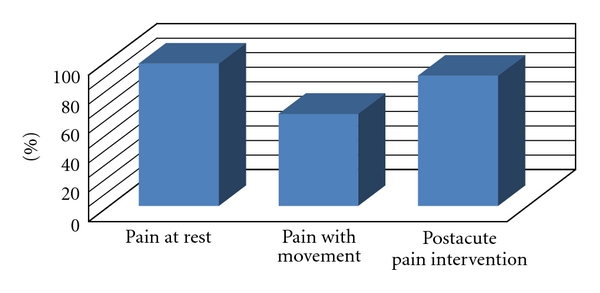
Percentage of responding hospitals that assess pain at rest, pain with movement, and reassess pain following an acute pain intervention.

Pain at rest was routinely measured in 105 hospitals (97.2%) (Figure 3). Pain with movement was measured in 68 hospitals (63%). Four hospitals did not respond to this question. Of 96 hospitals that routinely reassess and document the response to a pain control regimen intervention, 55 were academic (100% of academic hospitals), which is significantly greater (*P* < .05) when compared to 85% (34) of the private hospitals, and 54% (7) of VA hospitals. 

### 3.4. Postoperative Pain Protocols

**Figure 4:**
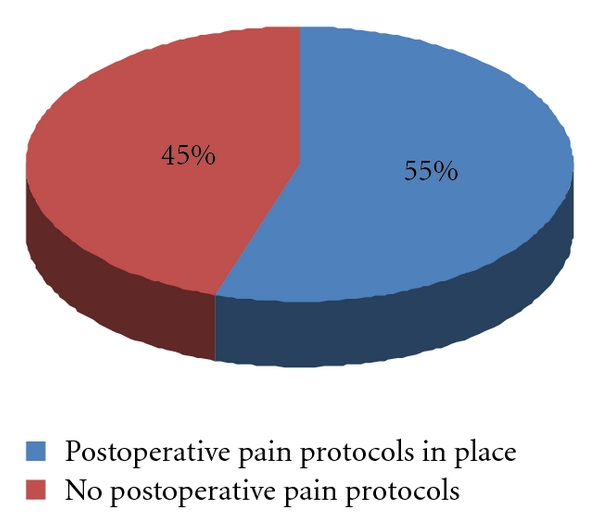
Percentage of responding hospitals with protocols for acute postoperative pain management.

Fifty-five percent (59 hospitals) of the responding institutions had formal written postoperative pain protocols (Figure 4). Hospitals with a formally organized APS reported a higher rate of following a previously prepared formal written postoperative pain protocol (89% of VA with an APS, 75% of academic, and 58% private hospitals (not significant when VA and academic hospitals were compared, but *P* < .05 when both academic and VA are compared to private hospitals)). Written postoperative pain protocols included requirements for vital sign monitoring, pain assessment and reassessment, and routine IV-PCA and epidural analgesia orders. Of these pain protocols, 52 hospitals (88.1%) included instructions on when and who to call for uncontrolled pain, 49 hospitals (83%) included pharmacological pain interventions, 37 hospitals (62.7%) included emergency “call orders”, and 34 hospitals (57.6%) included nonpharmacological pain interventions such as activities to improve sleep, reduce anxiety, or improve mood as well as repositioning, deep breathing, guided imagery, heat/cold application, massage therapy, physical or music therapy, and biofeedback. 

**Figure 5:**
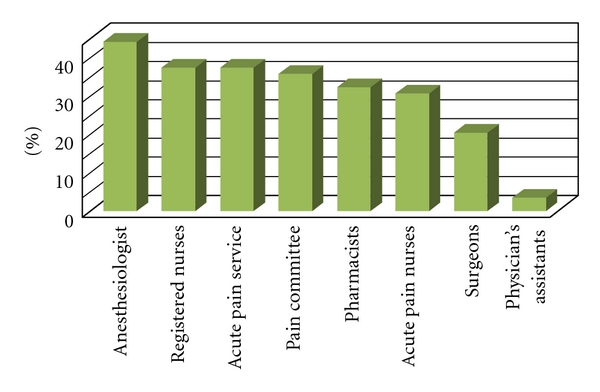
Percentage of each specialty or group participating in postoperative pain protocol development, Pain Comm (pain committee).

The postoperative pain protocols were normally developed in all institutions by using a multidisciplinary team approach. Primarily involved were anesthesiologists (44%), registered nurses (37.3%), the entire APS team (37.3%), multidisciplinary pain committees (35.6%), pharmacists (32.2%), advanced practice nurses (30.5%), surgeons (20.3%) and physician assistants (3.4%) (Figure 5). 

### 3.5. Postoperative Pain Therapy Followup

IV-PCA followup was conducted on a daily basis by surgeons (75%), anesthesiologists (37.1%), registered nurses (36.1%), the APS team (23.1%), physician assistants (16.7%), and advanced practice nurses (10.2%). One hospital did not reply. 

Epidural followup was conducted on a daily basis primarily by anesthesiologists (76%), the APS team (36.1%), advanced practice nurses (19.4%), registered nurses (16.7%), surgeons (4.6%) and physician assistants (4.6%). Five hospitals did not reply. 

**Figure 6:**
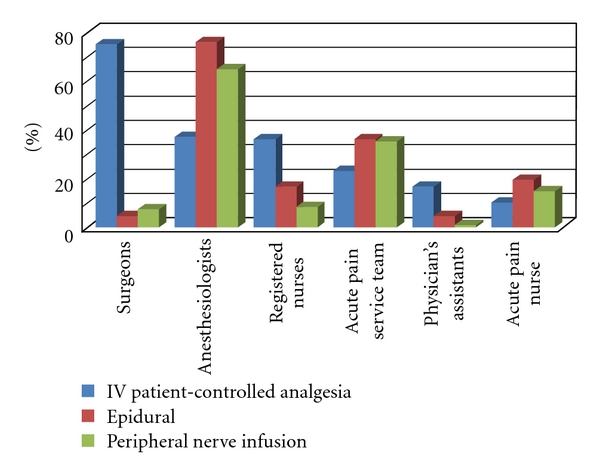
Percentage of each specialty managing different modalities for postoperative pain.

Peripheral nerve block infusion followup was conducted on a daily basis by anesthesiologists (64.8%), the APS team (35.2%), advanced practice nurses (14.8%), registered nurses (8.3%), surgeons (7.4%) and physician assistants (0.9%). Nine hospitals did not reply (Figure 6). 

### 3.6. Role of RN in IV-PCA Management

Under routine postoperative orders (written protocols), the RN could administer IV-PCA boluses for uncontrolled pain in 72.2% of the hospitals surveyed. The RN could adjust IV-PCA pump settings within certain parameters in 57.4% of the hospitals. In hospitals without IV-PCA clinician bolus orders, the surgeon or APS would bolus the IV-PCA or make rate adjustments while on rounds or when called by the staff RN. 

### 3.7. Role of RN in Epidural Management

Under routine postoperative orders, the RN could administer epidural boluses for uncontrolled pain in 25% of the hospitals surveyed. The RN could adjust epidural pump settings within certain parameters in 39.8% of the hospitals. In the remaining institutions where epidural analgesia was used, the APS or anesthesiologists would bolus the epidural or make infusion adjustments. 

### 3.8. Role of RN in Peripheral Nerve Block Infusion Management

**Figure 7:**
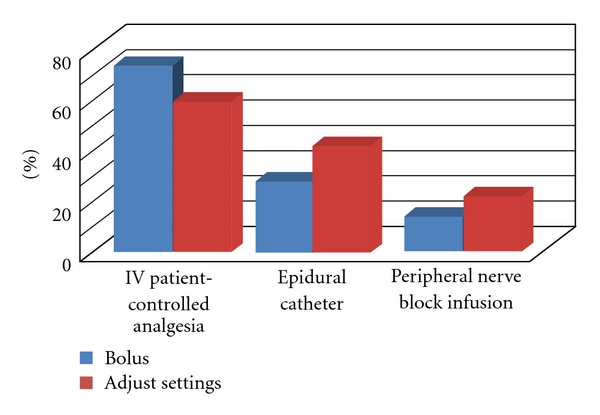
Percentage of responding institutions permitting RN bolus and titration of IV-PCA and epidural and peripheral nerve block infusions.

Under postoperative orders, the RN could administer peripheral nerve block infusion boluses in 12% of the hospitals surveyed. The RN could adjust PNB infusion pump settings within certain parameters in 19.4% of the hospitals. In the remaining institutions where PNB were used, the APS or anesthesiologist would bolus with a local anesthetic or make PNB infusion rate adjustments (Figure 7). 

### 3.9. Criteria for Pain Improvement

**Figure 8:**
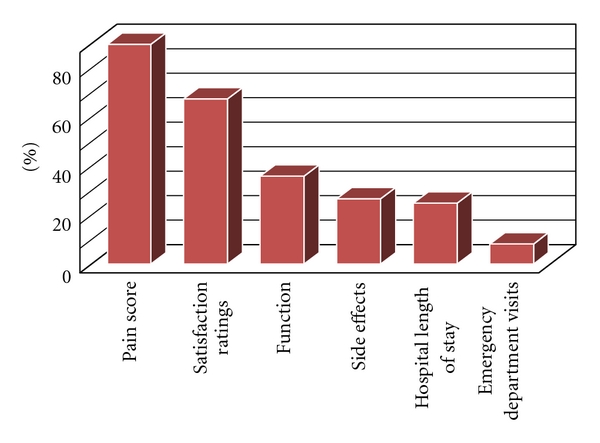
Percentage of specific measurements used to evaluate postoperative pain management effectiveness by responding institutions, LOS (length of stay).

Ninety percent (97 of 108) of the responding hospitals measured improvement in pain management by improved scores on a numeric rating scale. Ninety eight percent of the university/academic hospitals measured improvement in pain management by improved pain scores as well as 82% of private hospitals (not significant when compared to academic) and 77% of VA hospitals (*P* < .05, when compared to academic). Others measured improvement by higher patient satisfaction ratings (67.6%), improved functional ability (36.1%), fewer side effects from treatment (26.8%), and shorter hospital lengths of stay (25%). Eight percent of the hospitals measured improvement in terms of fewer emergency room visits for unrelieved postoperative pain. One hospital rated improvement in terms of fewer phone calls to administrators. Four hospitals did not answer this question (Figure 8). 

### 3.10. “On-Call” Coverage for Postoperative Pain Management

**Figure 9:**
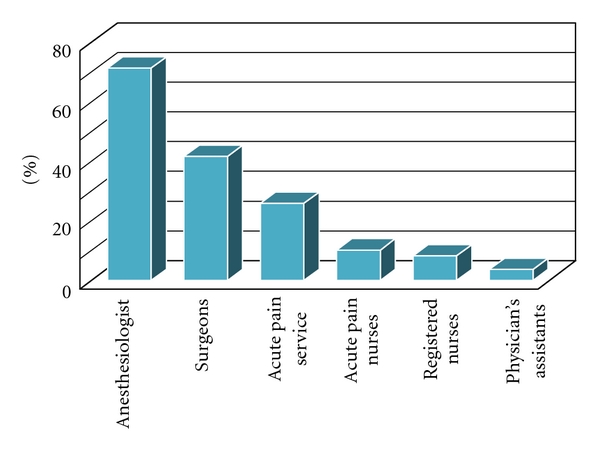
Percentage of specialty groups providing “on-call” coverage for postoperative pain.

**Figure 10:**
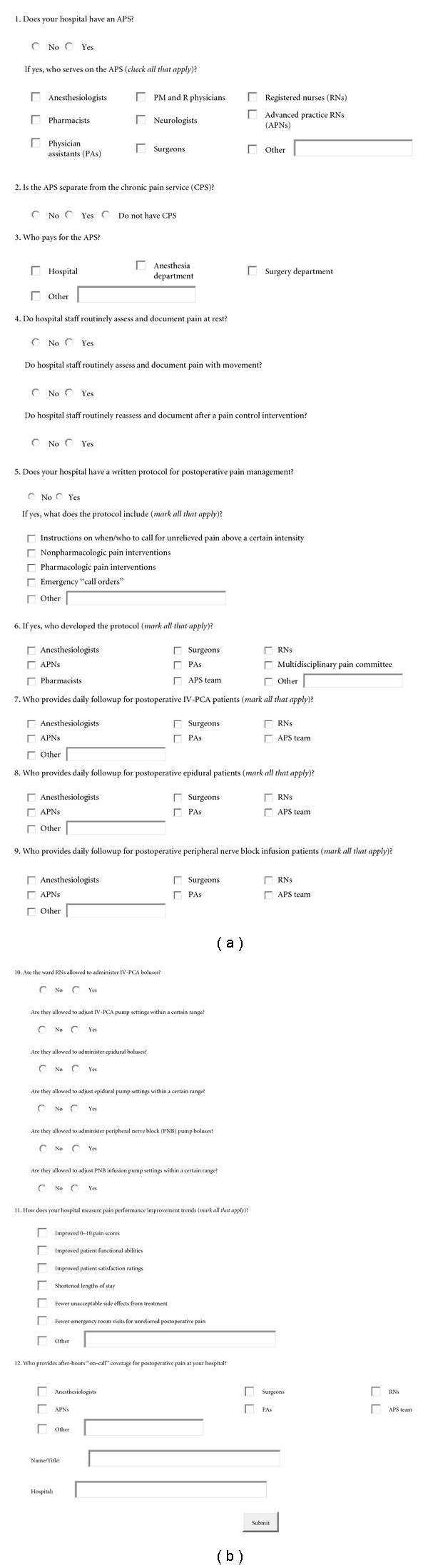
Acute pain service hospital survey.

Provision of after-hours “on-call” coverage for postoperative pain was most often provided by the following groups: anesthesiologists, anesthesia residents or fellows (71.3%), surgeons or surgery residents (41.7%), APS team (25.9%), advanced practice RN's including certified RN anesthetists (10.2%), RN's (8.3%), and physician assistants (3.7%) (Figure 9). 

## 4. Discussion

Since the initial reports [9–11], there are only a few studies evaluating the structure and function of APS [3, 4]. However, the current status of APS in the US remains limited although there are a few reports from Canada, Austalia, and Europe [3, 12–15]. This paper demonstrates that an organized APS with written protocol adherence is more common in academic/university hospitals than is present in either private nonteaching or VA hospitals. 

It is important to realize that this study has some limitations including a relatively low response rate from all classes of hospitals. It is possible that many hospitals did not respond because they do not have an APS. The responders may have been more likely those who felt that they had optimal APS, likely explaining why the results showed a higher. Another limitation may be related to the way questions were framed. Therefore, the information gathered may be skewed. Nevertheless, despite these limitations, this study provides valuable insight that should provide guidance towards improved functioning of an APS and improved pain management in hospitalized surgical patients.

This survey confirms the previous reports of significant variations in the structure and function [3, 4]. Although the number of hospitals offering an APS has increased in academic hospitals, the presence APS in nonacademic hospitals remains low. This may be due to the lack of funding allocated towards APS. This survey indicates that the APS is primarily funded and staffed through the institutional anesthesia departments. When the APS is funded by the hospital at large, the primary APS members continue to be anesthesiologists, combined with APNs and RNs. The expertise of anesthesiologists in pain management explains their significant involvement in APS. 

As indicated in this survey, a majority of patients receiving IV-PCA, which is the most commonly used analgesic technique in hospitalized patients, are followed by surgeons without the involvement of the APS. However, as suggested by this survey, surgeons appear to play a limited role in APS. In absence of surgical involvement, it is possible that involvement of nurses with special training in current concepts of postoperative pain management and implementation of pain protocols (e.g., pain resource nurses) may be a practical approach to reducing inadequate pain relief and improving patient satisfaction. Delegation of predefined pain control options to nurses on the surgical ward enables them to initiate pain therapy in a timely manner and adjust the regimen to meet individual patient needs. However, this survey found that nurses were less often directed to adjust IV-PCA settings and even fewer protocols direct nurses about adjustments in epidural analgesia and peripheral nerve blockade therapy. One of the functions of an APS should be to educate the nurses interested in participating in the pain management program. A specialized-nurse-based anesthesiologist supervised APS may be cost beneficial [3], although there are no such studies supporting this contention. 

Another important information from this survey was related to the lack of written protocols in 45% of the hospitals. One of the major functions of APS is to develop clinically useful protocols based on published evidence, even if there is no direct involvement in patient care (e.g., for patients receiving IV-PCA). This should ensure a more consistent standard of care as well as provide instructions on diagnosis (through appropriate monitoring) and treatment of adverse events. In addition, the protocols would provide instructions on when and who to call for unrelieved pain and instructions for management of special group of patients (e.g., opioid tolerant patients). 

It has become increasingly clear that use of evidence-based approach to patient care improves outcome and reduces adverse events [16]. However, most postoperative pain management guidelines are developed from combining evidence from multiple surgical procedures (ASA Guidelines; 17). However, different surgical procedures produce different pain characteristics that require different treatment approaches. In addition, the risks and benefits of different analgesic techniques differ between surgical procedures. Therefore, procedure-specific, evidence-based guidelines are necessary to provide optimal patient care and improved outcomes [16–18]. Procedure-specific systematic reviews and recommendations for some surgical procedures are available online (http://www.postoppain.org/).

Another important finding of this study is inconsistency in pain assessment with activity and reassessment after a pain intervention. Reassessment gives the clinician the tools needed to individualize treatment based on efficacy, side effects, and improvement in pain intensity and patient function. Only 63% of responders evaluated pain scores on activity in spite of good evidence that pain control with activity (e.g., physical therapy, cough, and deep breathing exercises) is an important factor in postoperative rehabilitation and should be evaluated regularly. 

This also indicates that most of the hospitals used only pain scores to evaluate improvement in pain management, and only a few hospitals used improved functional ability or side effects from analgesic therapy. However, it is increasingly clear that pain intensity scores alone should not be used to manage postoperative pain. Recent studies have reported increased use of opioids and opioids-related side effects since the introduction of “pain as a fifth vital sign” campaign by the Joint Commission [19–22]. Therefore, there should be increased emphasis on use of patient outcomes to guide pain therapy rather than pain scores alone.

## 5. Conclusions

In summary, the structure and function of APS in the US hospitals vary significantly, with university/academic institutions being more likely to offer these services than private or VA hospitals. There appears to be lack of consensus regarding the use of pain management of protocols as well as optimal outcome measures for assessing postoperative pain therapy. Further research is required to evaluate the impact of evidence-based procedure-specific pain management protocols and assessment of pain on activity as well as increased role of nurses on overall pain management and postoperative outcome. Finally, the cost benefit of hospital-based APS also needs to be critically evaluated.
